# Fatty Acid Fingerprints and Hyaluronic Acid in Extracellular Vesicles from Proliferating Human Fibroblast-like Synoviocytes

**DOI:** 10.3390/ijms23105613

**Published:** 2022-05-17

**Authors:** Anne-Mari Mustonen, Tommi Paakkonen, Johanna Matilainen, Kirsi Rilla, Reijo Käkelä, Marjo Malinen, Piia Takabe, Sanna Oikari, Janne Capra, Sanna P. Sihvo, Pauliina Ryökäs, Petteri Nieminen

**Affiliations:** 1 Institute of Biomedicine, School of Medicine, Faculty of Health Sciences, University of Eastern Finland, P.O. Box 1627, FI-70211 Kuopio, Finland; tommi.paakkonen@uef.fi (T.P.); johanna.matilainen@uef.fi (J.M.); kirsi.rilla@uef.fi (K.R.); piia.takabe@uef.fi (P.T.); sanna.oikari@uef.fi (S.O.); pauliina.ryokas@uef.fi (P.R.); petteri.nieminen@uef.fi (P.N.); 2 Department of Environmental and Biological Sciences, Faculty of Science and Forestry, University of Eastern Finland, P.O. Box 111, FI-80101 Joensuu, Finland; marjo.malinen@uef.fi; 3 Molecular and Integrative Biosciences Research Programme, Faculty of Biological and Environmental Sciences, University of Helsinki, P.O. Box 65, FI-00014 Helsinki, Finland; reijo.kakela@helsinki.fi (R.K.); sanna.sihvo@helsinki.fi (S.P.S.); 4 Helsinki University Lipidomics Unit (HiLIPID), Helsinki Institute of Life Science (HiLIFE) and Biocenter Finland, P.O. Box 65, FI-00014 Helsinki, Finland; 5 Cell and Tissue Imaging Unit, Institute of Biomedicine, School of Medicine, Faculty of Health Sciences, University of Eastern Finland, P.O. Box 1627, FI-70211 Kuopio, Finland; janne.capra@uef.fi

**Keywords:** arthritis, extracellular vesicle, fatty acid, fibroblast-like synoviocyte, hyaluronan, joint disease

## Abstract

Extracellular vesicles (EVs) function as conveyors of fatty acids (FAs) and other bioactive lipids and can modulate the gene expression and behavior of target cells. EV lipid composition influences the fluidity and stability of EV membranes and reflects the availability of lipid mediator precursors. Fibroblast-like synoviocytes (FLSs) secrete EVs that transport hyaluronic acid (HA). FLSs play a central role in inflammation, pannus formation, and cartilage degradation in joint diseases, and EVs have recently emerged as potential mediators of these effects. The aim of the present study was to follow temporal changes in HA and EV secretion by normal FLSs, and to characterize the FA profiles of FLSs and EVs during proliferation. The methods used included nanoparticle tracking analysis, confocal laser scanning microscopy, sandwich-type enzyme-linked sorbent assay, quantitative PCR, and gas chromatography. The expression of hyaluronan synthases 1–3 in FLSs and HA concentrations in conditioned media decreased during cell proliferation. This was associated with elevated proportions of 20:4n-6 and total n-6 polyunsaturated FAs (PUFAs) in high-density cells, reductions in n-3/n-6 PUFA ratios, and up-regulation of cluster of differentiation 44, tumor necrosis factor *α*, peroxisome proliferator-activated receptor (*PPAR*)-*α*, and *PPAR-γ*. Compared to the parent FLSs, 16:0, 18:0, and 18:1n-9 were enriched in the EV fraction. EV counts decreased during cell growth, and 18:2n-6 in EVs correlated with the cell count. To conclude, FLS proliferation was featured by increased 20:4n-6 proportions and reduced n-3/n-6 PUFA ratios, and FAs with a low degree of unsaturation were selectively transferred from FLSs into EVs. These FA modifications have the potential to affect membrane fluidity, biosynthesis of lipid mediators, and inflammatory processes in joints, and could eventually provide tools for translational studies to counteract cartilage degradation in inflammatory joint diseases.

## 1. Introduction

Extracellular vesicles (EVs) are nano- and micro-sized membrane-bound particles released by virtually all cell types [1,2]. They are often classified as exosomes (EXOs), microvesicles (MVs), and apoptotic bodies (ABs) that are formed by different modes of biogenesis and overlap in size. EXOs (diameter generally reported as 30–250 nm) and MVs (100–1000 nm) have endosomal or plasma membrane origins, while ABs that tend to be larger (>1000 nm) are formed by plasma membrane blebbing during programmed cell death. EVs act as conveyors of bioactive lipids and other molecules and can modulate the gene expression patterns and behavior of target cells. Research on the physiological and pathological roles of EVs has become very active, and EVs have been suggested to reflect and affect different inflammatory conditions, including chronic age-associated joint diseases osteoarthritis (OA) and rheumatoid arthritis (RA). Joint pathology can be associated with altered EV concentrations and cargo in synovial fluid (SF). SF EVs could propagate inflammation and cartilage degradation in inflamed joints by transporting and enhancing the production of inflammatory factors and proteinases that degrade the cartilage extracellular matrix. EVs may also induce beneficial effects on joint health, for instance, by transporting hyaluronic acid (HA) that provides boundary lubrication for articular cartilage [3].

SF HA concentrations and HA molecular weight are reduced in inflamed joints [4], and HA administrations can have a beneficial influence on joint health, including improved lubrication, chondroprotection, and anti-inflammatory effects [5]. The HA matrix around fibroblast-like synoviocytes (FLSs) appears to function as a natural sponge for EVs as it can facilitate their internalization [6]. To date, the HA coat and lipid composition of EV membranes have not been the focus of intensive research, even though EV lipids have a structural role, they can influence membrane fluidity and curvature, and contain signaling molecules with a great potential to affect EV binding and uptake, and to modify the behavior of recipient cells [7,8]. EV lipids and enzymes involved in their metabolism also affect the formation and release of EVs and, in addition, bioactive lipids can influence cellular HA synthesis [9].

In comparison to parent cells, EVs have been documented to be enriched in cholesterol, sphingomyelin (SM), glycosphingolipids, and phosphatidylserine (PS), whereas the levels of phosphatidylcholine (PC) and phosphatidylinositol (PI) are often lower [8,10]. Ether lipids can potentially affect EV rigidity, membrane fusion, and stability in the extracellular space [7]. Regarding fatty acids (FAs), EV membrane lipids have been reported to accumulate 14–18C saturated FAs (SFAs), 18:1, 24:0, and 24:1, and to have reduced unsaturation [7,10,11]. In vitro manipulation of parent cells with polyunsaturated FAs (PUFAs) has been shown to increase the levels of 18:1 and di-PUFA-phospholipids (PLs) in EVs with potential implications in the biosynthesis of PUFA-derived lipid mediators (LMs) [12]. EVs are known to transport LMs, such as prostaglandin E_2_ (PGE_2_) and 15-deoxy-∆^12,14^-prostaglandin J_2_, and the enzymatic machinery required for their synthesis, including phospholipase A_2_ and cyclooxygenase (COX)-2 [13]. Specialized pro-resolving mediators and their precursors have also been detected in EVs, suggesting possible roles in the resolution of inflammation [14].

FLSs play central roles in inflammation, pannus formation, and joint destruction in arthropathies [15,16], and EVs have recently emerged as potential mediators of these effects [2]. It was demonstrated that EVs released by immune cells increased the synthesis of inflammatory mediators and cartilage-degrading proteinases by OA and RA synovial fibroblasts [17], and similarly, EVs in RA SF stimulated the release of cytokines and chemokines by FLSs [18,19]. Furthermore, EXOs from interleukin (IL)-1*β*-stimulated synovial fibroblasts increased the expression of OA-related genes in articular chondrocytes [20]. In contrast, EVs from adipose tissue mesenchymal stem cells (MSCs) reduced the expression of pro-inflammatory factors in a chronic model of FLS inflammation [6]. Inflammatory conditions can increase the synthesis of HA and PLs by OA FLSs [21,22].

To the best of our knowledge, the secretion of HA-containing EVs (HA–EVs) by FLSs has not been a focus of intensive research. Neither have the FA profiles of FLS EVs been examined in detail, while it is known that EVs from dermal fibroblasts can be enriched with SFAs and PUFAs compared to parent cells [23]. As FLS EVs may alleviate OA or contribute to its pathogenesis, it would be important to characterize the FA composition of these particles. FAs have the potential to induce a plethora of beneficial and detrimental actions on different joint tissues [24], and their composition in EVs can influence the stability of EV membranes as well as reflect the availability of precursors for transcellular biosynthesis of LMs [23]. The aims of the present study were (*i*) to examine the HA and EV secretion by normal FLSs, (*ii*) to determine the expression of related genes, and (*iii*) to characterize the FA composition of FLS EVs, all in a temporal pattern during cell proliferation. It was hypothesized that (*i*) HA synthesis and secretion would decrease during cell proliferation, (*ii*) EV release would be stimulated during cell growth, and that (*iii*) FA transfer from FLSs into EVs would be selective.

## 2. Results

### 2.1. Imaging of FLSs and Their HA and EV Release

**Figure 1:**
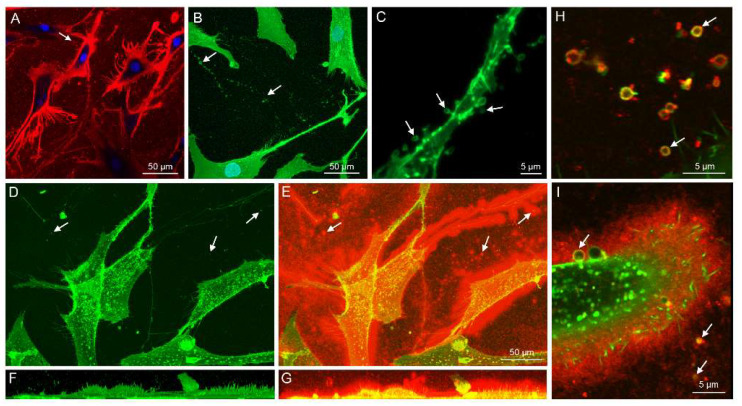
Confocal optical section of live fibroblast-like synoviocytes stained with Alexa Fluor 568-labeled hyaluronan (HA) binding complex (HABC) and NucBlue (pseudocolored red and blue, respectively, panel (**A**)). Fixed and immunostained cells with CD44 antibody (pseudocolored green) as maximum intensity projection from a stack of images (panel (**B**)) and the same culture imaged with super-resolution to indicate extracellular vesicle (EV) budding from the membrane of a single cell (panel (**C**) (maximum intensity projection from a stack of images). Live cells labeled with CellMask Deep Red plasma membrane stain (pseudocolored green, panel (**D**) and merged with Alexa Fluor-labeled HABC to detect HA (pseudocolored red, panel (**E**) as maximum intensity projections from a stack of images. The corresponding side views of panels (**D**) and (**E**) are shown in panels (**F**) and (**G**), respectively. Super-resolution (Airyscan^®^) optical sections from the same cultures shown in panels (**D**–**G**) indicate single EVs of variable size that are attached to the bottom of the culture plate (pseudocolored green, panel (**H**)) and a more detailed structure of the HA coat around the plasma membrane with budding EVs (pseudocolored red, panel (**I**)). Arrows in all panels indicate EVs.

FLSs were cultured for up to eight passages, and they exhibited homogenous fibroblastic (spindle-shaped) morphology (Figure 1A) with no change in cell viability. A thick pericellular HA coat (5–15 μm) was observed around cells (Figure 1A,E,I). The release of individual EVs from the plasma membrane was visualized from live FLSs with confocal laser scanning microscopy (CLSM) (Figure 1A–I). Also, EVs that were left behind by migrating cells could be observed attached to the bottom of ibidi chambers (Figure 1H).

**Figure 2:**
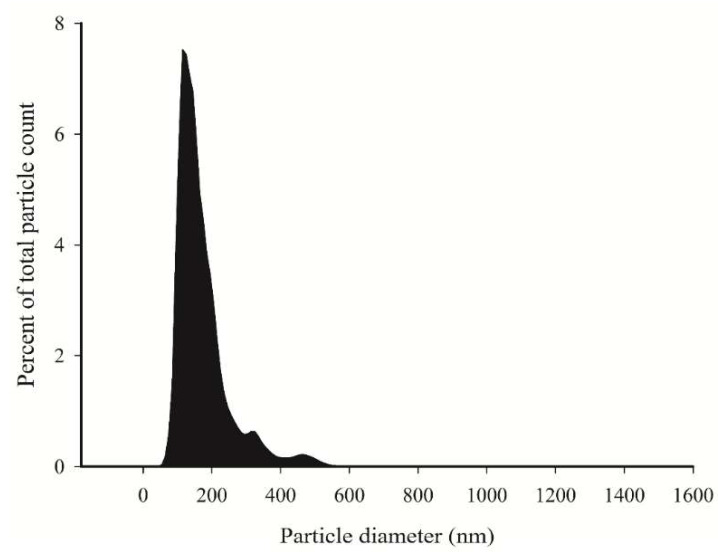
The size distribution of nano-sized particles in the 5 μm-filtered conditioned media of fibroblast-like synoviocytes (*n* = 5) determined by nanoparticle tracking analysis.

**Figure 3:**
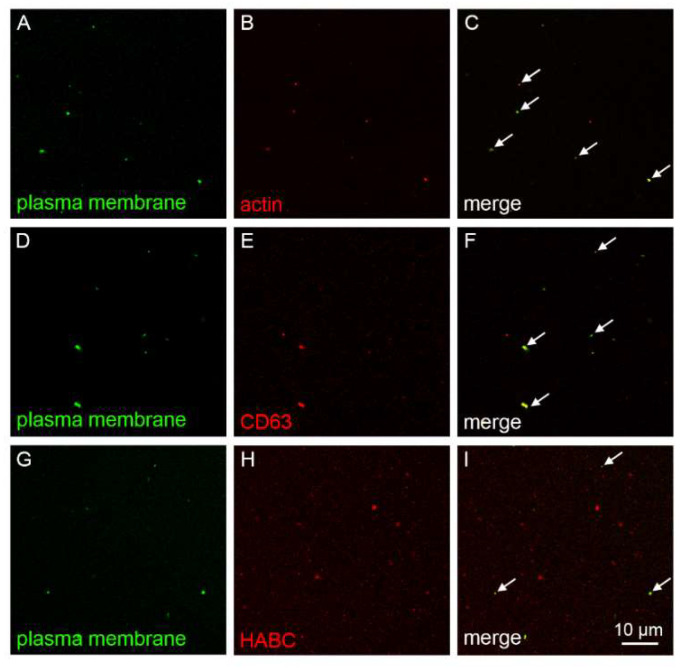
Characterization of extracellular vesicles isolated by ultracentrifugation from fibroblast-like synoviocyte culture medium and stained with CellMask Deep Red plasma membrane stain (pseudocolored green, panels (**A**,**D**,**G**)), combined with Alexa Fluor 594-labeled phalloidin to detect actin (panel (**B**)), Alexa Fluor 488-labeled CD63 (panel (**E**)), and Alexa Fluor 568-labeled hyaluronan (HA) binding complex (HABC) to detect HA (panel (**H**)), all pseudocolored red. Merged images are shown in panels (**C**,**F**,**I**), correspondingly. Arrows in merged images indicate examples of co-localization of the stains.

**Figure 4:**
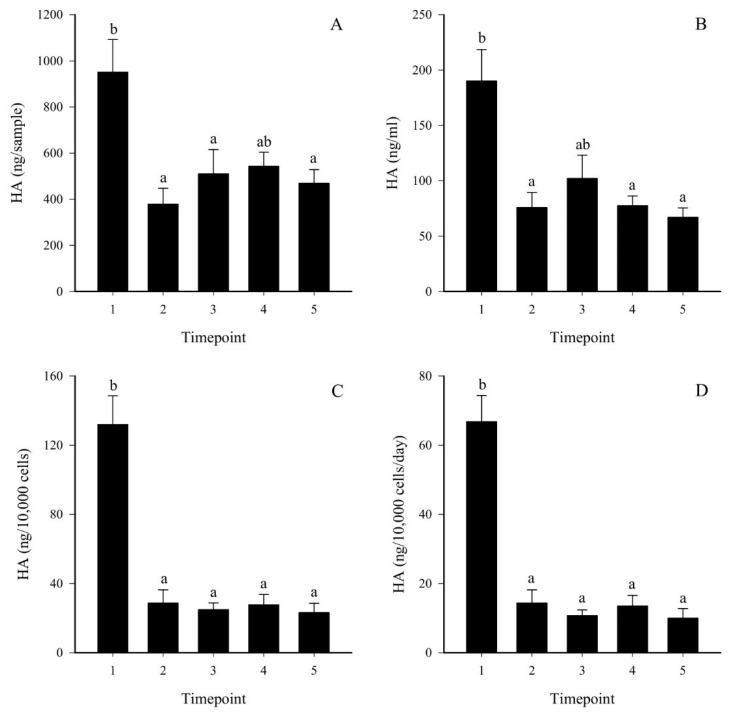
Temporal changes in hyaluronan (HA) concentrations in conditioned medium of fibroblast-like synoviocytes (mean + SE), represented as ng/sample (panel (**A**)), ng/mL (panel (**B**)), ng/10,000 cells (panel (**C**)), and ng/10,000 cells/day (panel (**D**)). Timepoint 1 = lag phase, timepoints 2–3 = proliferating cells, timepoints 4–5 = high-density cells. Means with dissimilar letters indicate significant differences between timepoints (Kruskal–Wallis ANOVA, *p* < 0.05).

After EV collection from FLS-conditioned media, nanoparticle tracking analysis (NTA) and CLSM were utilized to observe the number and size distribution of EVs. The main population was <200 nm in diameter as determined by NTA (Figure 2). With CLSM, the average EV diameter was higher, 1530 ± 48 nm, indicating that they consisted of a different subpopulation when visualized with this method (Figure 3). The count of larger EVs was higher at timepoints 1–2 compared to timepoint 5, while the EV area and diameter increased during cell growth (Table 1). The temporal reduction in the EV count was also evident when expressed per cell. The medium HA concentrations reduced between timepoints 1 and 2 and remained low at timepoints 3–5 (Figure 4). According to the ComDet analysis, most EVs contained HA-fluorescence, i.e., they were transporting HA (Figure 1H–I, Figure 3G–I), and there was also a temporal decrease in HA–EV counts (Table 1).

### 2.2. Real-Time Quantitative PCR (qPCR) Results of FLSs

**Figure 5:**
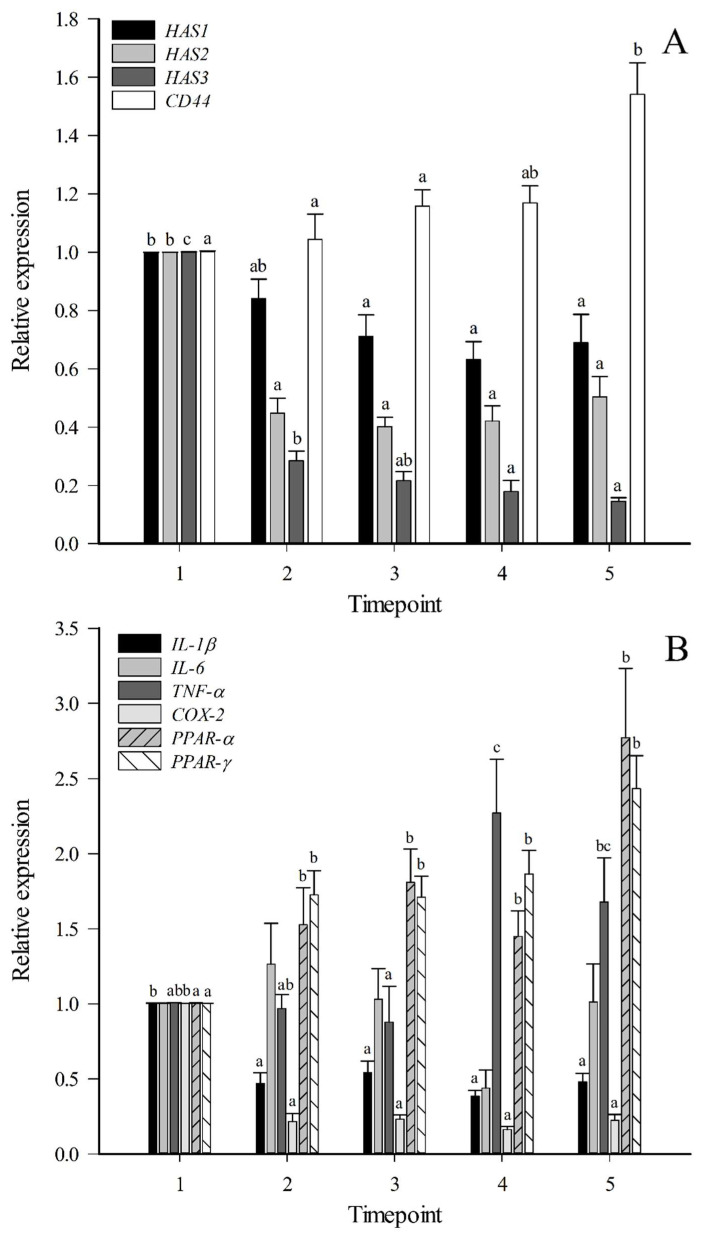
Temporal changes in the expression of selected genes in fibroblast-like synoviocytes (mean + SE). Real-time quantitative PCR was used to determine the ratio of the mRNA expression of the genes of interest relative to the control gene ribosomal protein lateral stalk subunit P0. Panel (**A**) represents *HAS1–3* = hyaluronan synthase 1–3 and *CD44* = cluster of differentiation 44. Panel (**B**) shows *IL-1β/6* = interleukin-1*β*/6, *TNF-α* = tumor necrosis factor *α*, *COX-2* = cyclooxygenase 2, and *PPAR-α/γ* = peroxisome proliferator-activated receptor *α*/*γ*. Timepoint 1 = lag phase, timepoints 2–3 = proliferating cells, timepoints 4–5 = high-density cells. Means with dissimilar letters indicate significant differences between timepoints (Kruskal–Wallis ANOVA, *p* < 0.05).

Hyaluronan synthase (*HAS*)*2* was the major *HAS* mRNA isoform expressed in FLSs during proliferation, followed by *HAS1* and *HAS3* (Figure S1). The mRNA expression of all isoforms decreased at timepoints 3–5 (*HAS1*) or 2–5 (*HAS2*, *HAS3*) compared to timepoint 1 (Figure 5A). *CD44*, encoding a major cell surface receptor of HA, was up-regulated at timepoint 5. Other examined genes with temporal reduction in the mRNA expression included *IL-1β* and *COX-2*, while tumor necrosis factor (*TNF*)-*α*, peroxisome proliferator-activated receptor (*PPAR*)-*α*, and *PPAR-γ* showed increased expression (Figure 5B). The mRNA expression of *CD44*, *TNF-α*, *PPAR-α*, and *PPAR-γ* correlated positively with the number of days from passage, confluence, and/or FLS count (r_s_ = 0.340–0.677, *p* ≤ 0.001–0.032), whereas the correlations were negative for *HAS1–3*, *IL-1β*, *IL-6*, and *COX-2* (r_s_ = −0.337–−0.801, *p* ≤ 0.001–0.048). There were no differences in the *IL-6* mRNA expression between timepoints.

### 2.3. Fatty Acid Signatures (FASs) of Different Sample Types

**Figure 6:**
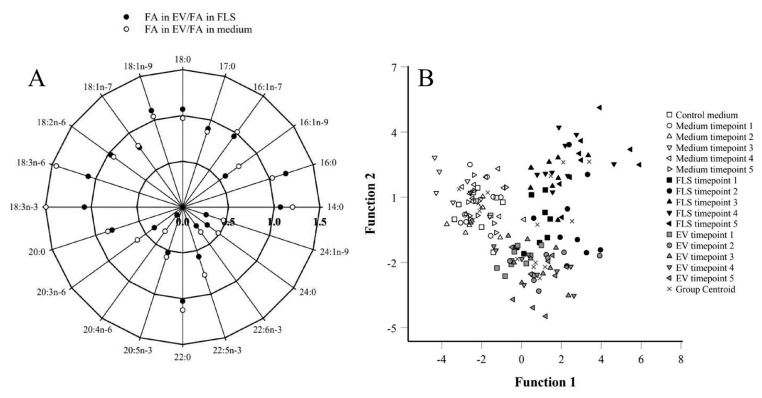
The plot of the fractionation coefficients of selected fatty acids (FAs) in extracellular vesicles (EVs) vs. fibroblast-like synoviocytes (FLSs) (●) or in EVs vs. FLS-conditioned media (○) (panel (**A**)). Values <1.0 indicate that the mol-% of a given FA is higher in FLSs or media than in EVs and values >1.0 indicate that the mol-% is higher in EVs than in FLSs or media. Discriminant analysis of FA proportions in control and FLS-conditioned media, FLSs, and EVs collected during 5 different timepoints: 1 = lag phase, 2–3 = proliferating cells, 4–5 = high-density cells (panel (**B**)). Function 1 (on *x*-axis) explained 58.5% of the variance in the dataset and function 2 (on *y*-axis) 41.5% of the variance. White symbols = media, black symbols = FLSs, grey symbols = EVs.

The detailed FA composition of the samples is presented in Table S1. The proportions of 14:1n-5 were lower in FLS-conditioned media, while those of 16:1n-9 were higher compared to control media. FLSs had lower percentages of 16:0, 18:0, 18:1n-9, and total SFAs, as well as reduced total average chain lengths (TACLs) compared to conditioned media. In addition, FLSs had higher proportions of most other FAs, including 22–24C monounsaturated FAs (MUFAs), total n-3 and n-6 PUFAs, and total dimethyl acetals (DMAs), and higher unsaturated FA (UFA)/SFA ratios and double bond indices (DBIs). The FAs with increased percentages in the EV fraction compared to FLSs were 16:0, 18:0, and 18:1n-9 (Figure 6A). Total SFAs and n-3/n-6 PUFA ratios were also elevated. Most other FAs, especially 22–24C MUFAs and 20–22C PUFAs, were reduced in the EV fraction. Regarding the FA sums and ratios, n-3 and n-6 PUFAs, DMAs, UFA/SFA ratios, DBIs, and TACLs were also higher in FLSs. In the discriminant analysis, functions 1–2 accounted for 100% of the variance in the dataset. The samples clustered in 3 groups (i.e., both medium types together, FLSs, and EV fraction) that were mostly separated from each other (Figure 6B). The discriminant function 1 depicted on the horizontal axis separated the media from the cells and EVs, and the variables mainly responsible for this included DMA 18:0, DMA 16:0, 15:0ai, 18:3n-3, and 14:0. The cells and EVs showed separation by the discriminant function 2 on the vertical axis by FAs, such as 20:3n-6, 16:0, 15:0i, and 24:0. The analysis classified 95.8% of samples correctly into their respective groups.

### 2.4. Temporal Changes in the FASs

**Figure 7:**
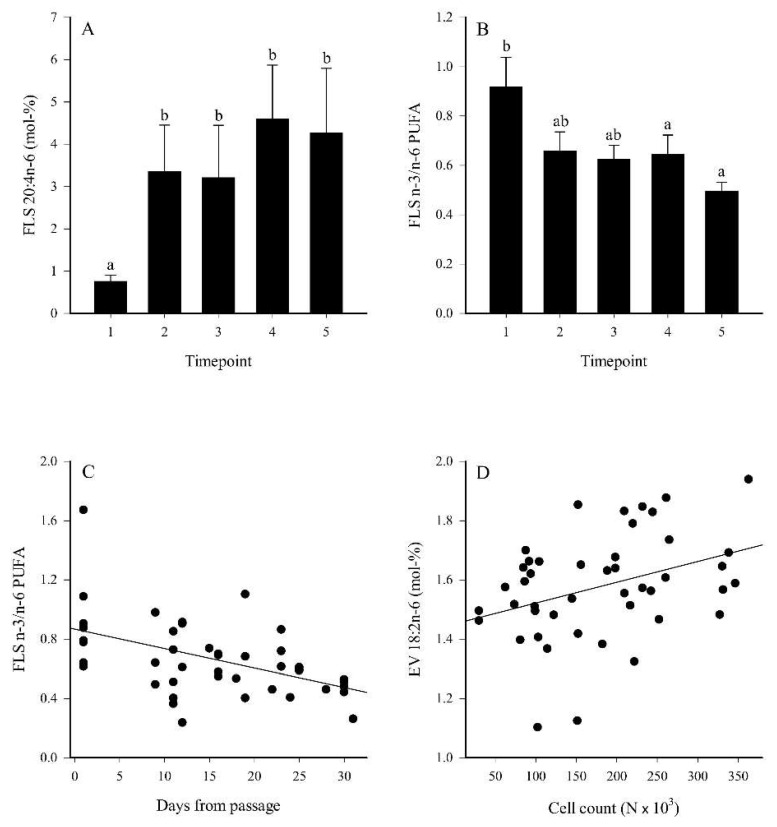
Temporal changes in the proportions of 20:4n-6 (panel (**A**)) and ratios of n-3/n-6 polyunsaturated fatty acids (PUFAs) (panel (**B**)) in fibroblast-like synoviocytes (FLSs) (mean + SE), and correlations between n-3/n-6 PUFA ratios in FLSs and the number of days from passage (r_s_ = –0.481, *p* = 0.001; panel (**C**)) and between 18:2n-6 proportions in extracellular vesicles (EVs) and FLS counts (r_s_ = 0.342, *p* = 0.021; panel (**D**)). Timepoint 1 = lag phase, timepoints 2–3 = proliferating cells, and timepoints 4–5 = high-density cells. Dissimilar letters above bars indicate significant differences between timepoints (Kruskal–Wallis ANOVA, *p* < 0.05).

**Figure 8:**
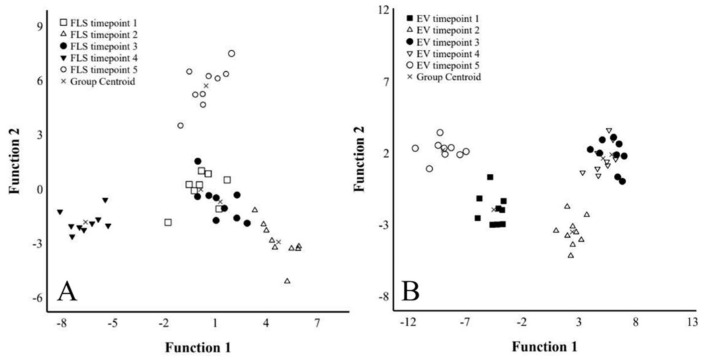
Discriminant analysis of fatty acid proportions in fibroblast-like synoviocytes (FLSs) and extracellular vesicles (EVs) collected during 5 different timepoints: 1 = lag phase, 2–3 = proliferating cells, and 4–5 = high-density cells. (Panel (**A**)) presents the FLS data, in which function 1 explained 45.1% of the variance in the dataset and function 2 accounted for 29.7% of the variance. (Panel (**B**)) presents the EV fraction, in which functions 1–2 explained 92.1% of the variance in the dataset.

The proportions of 20:4n-6 in FLSs were lower at timepoint 1 compared to timepoints 2–5 (Figure 7A), and this was also reflected in the total n-6 PUFA sums (Figure S2). The n-3/n-6 PUFA ratios decreased at timepoints 4–5 compared to timepoint 1 (Figure 7B). Levels for 24:0 were elevated at timepoints 4–5 compared to timepoints 1–2, and the percentages of 17:1n-8 were higher at timepoints 3–4 compared to the other measurements (Figure S2). The proportions of 20:3n-6, 20:4n-6, 24:0, and total n-6 PUFAs correlated positively with the number of days from passage, confluence, and/or FLS count (r_s_ = 0.306–0.507, *p* ≤ 0.001–0.043), whereas the correlations were negative for 14:0, total MUFAs, and n-3/n-6 PUFA ratios (r_s_ = −0.326–−0.481, *p* = 0.001–0.031; Figure 7C). According to the discriminant analysis, the FLS FASs of timepoints 1 and 3 clustered together and the FAS of timepoint 2 aligned close to these groups, whereas timepoints 4–5 clustered separately from each other and from the other FLS samples (Figure 8A). Function 1, which explained 45.1% of the variance in the dataset, separated timepoints 2 and 4 from the others, and the FAs mainly responsible for this were 24:1n-9 and 16:1n-5. In particular, timepoint 5 was separated by function 2, explaining 29.7% of the variance, and the FAs with the highest separation power were 17:0ai, 14:1n-5, and 18:1n-7. The analysis classified 100% of samples correctly into their respective timepoints.

There were no statistically significant temporal alterations in the FA profiles of the EV fraction according to the Kruskal–Wallis analysis of variance (ANOVA). However, timepoints 3–4 clustered together in the discriminant analysis, whereas the other timepoints were clearly separate from each other (Figure 8B). Functions 1–2 explained 92.1% of the variance in the dataset. Function 1 separated timepoints 1 and 5 from the others, and 22:1n-7 had the largest separation power. In particular, timepoint 2 was separated by function 2 with 18:2n-6, 18:3n-6, 17:0ai, and 20:5n-3 as the most significant separating FAs. The analysis classified 100% of samples into the correct timepoint. Proportions of 18:2n-6 in the EV fraction correlated positively with confluence (r_s_ = 0.412, *p* = 0.005) and cell count (r_s_ = 0.342, *p* = 0.021; Figure 7D). There were no significant associations between the EV counts and the proportions of the main FAs or FA sums in the EV fraction.

## 3. Discussion

### 3.1. EV and HA Release and FLS Gene Expression

EVs were isolated from FLS-conditioned medium by ultracentrifugation and their characteristics validated by NTA and CLSM. They were positive for surface antigens CD44 and CD63, and for phalloidin that binds to filamentous actin, which is a cytosolic protein recovered in EVs [25]. These positive stainings were co-localized with CellMask plasma membrane stain and HA binding complex (HABC), verifying that FLSs secrete HA-transporting EVs [3]. EV size was variable in FLS-conditioned medium, and the subpopulations consisted of smaller EVs (most likely EXOs and MVs) by NTA and of larger EVs (MVs and ABs) when visualized by CLSM. Larger EVs were observed to directly bud from the plasma membrane, and they also attached to the bottom of ibidi chambers when the cells migrated. Similar to other methods for EV visualization [25], CLSM has limitations, but it can still be considered a useful supplement to EV studies. While it does not necessarily detect the smallest particles, CLSM can show phenomena, such as the association of EV membranes and HA-particles, and assess their potential significance in both the progress and amelioration of OA and related diseases.

The HA coat around FLSs is known to affect EV uptake to cells, which may be partially mediated by CD44 on the EV surface [6]. The present study documented a thick HA coat up to 15 μm around FLSs, and increased expression of *CD44* in high-density cells. All *HAS1–3* genes were expressed by FLSs, but *HAS2* was the most expressed isoform. Previously, *HAS1* was determined to be the predominant isoform in human synovial fibroblasts followed by *HAS2* and *HAS3* [26]. HAS3 is known to produce HA polymers of smaller size compared to the other isoforms [27]. It can be hypothesized that the thick HA coat could be related to the finding of *HAS2* as the dominant isoform, as it has previously been observed that the HA coat formed by HAS2 transfectants was significantly larger than that by HAS1 transfectants [27].

The temporal reduction in the medium HA concentration likely derived from the documented lower expression of *HAS1–3* during FLS proliferation. Decreased HA synthesis with increasing cell density has previously been observed for different fibroblast cell cultures [28]. IL-1*β* and TNF-*α* are among factors stimulating HA secretion by OA and RA synoviocytes [29], but in the present study, the expressions of these cytokines changed to opposite directions when the cell density increased. HA is known to suppress the proliferation of synoviocytes, but only at high concentrations of high-molecular-weight HA, such as those present in normal SF [30]. The HA levels in our culture medium were much lower, approximately 0.1 μg/mL, and highly unlikely to affect proliferation.

Cell type, density, and detachment of cells are among factors that can potentially affect the secretion of EVs in cultures [31]. The present study observed a temporal reduction in the EV and HA–EV counts in FLS-conditioned medium during cell proliferation. It was previously reported by Patel et al. [32] using several cell lines that EV production per cell was higher at lower seeding densities. We hypothesize that the stress caused by passaging could lead to increased EV release, and EV secretion could also be a compensatory mechanism for intercellular communication in low-density cells with fewer cell-to-cell contacts [32]. On the other hand, the decreased EV release by high-density cells may result from inhibition caused by the subsequently high number of cell-to-cell contacts [31]. EVs have been documented to promote cellular adhesion [33] and to affect proliferation [34,35]. FLS-derived EXOs stimulated chondrocyte proliferation and migration in association with cartilage repair [36], while bone marrow MSC-derived EVs inhibited RA-FLS proliferation and viability [37] and may, thus, influence pannus formation. These findings suggest that EVs of different origin may also induce beneficial effects on joint tissues. EVs could transport bioactive lipids and other molecules between cells and their FA cargo could participate in intercellular communication on inflammatory and resolution processes.

### 3.2. FLS FASs during Cell Proliferation

Knowledge about the molecular changes that occur during the culture of primary FLSs is limited [38]. While inflammatory factors can stimulate the proliferation of OA and RA synoviocytes [29], and synovial hyperplasia is considered essential for joint destruction in RA [15], the understanding of the mechanisms of normal FLS proliferation remains incomplete. Cell density can affect, for instance, lipid composition and intracellular trafficking, and it was previously noted in HEp-2 cells that the levels and species distribution of several lipids, such as diacylglycerols, phosphatidic acids, cholesterol esters, and lyso-PEs (phosphatidylethanolamines), were altered during culture for one to three days [39]. Different cancer cell lines have an increased demand for MUFAs during growth [40] and, regarding hepatocytes, MUFA-containing PCs were proposed as markers of cell proliferation [41]. In addition, FA profiles were documented to change during the differentiation of pre-adipocytes to mature adipocytes [42]. In this case, 16:0 and 16:1n-9 clearly increased, while 18:0, 20:4n-6, and 22:6n-3 decreased in proportion.

Similar to studies on EV release by FLSs, research on the temporal changes in FLS lipid composition during cell proliferation is in the early stages. The FA modifications of cells can affect, for instance, membrane fluidity, properties of membrane-bound receptors, eicosanoid production, and growth [43]. The present study documented elevated proportions of 20:4n-6, 24:0, and total n-6 PUFAs in proliferating cells and a simultaneous decrease in n-3/n-6 PUFA ratios. 20:4n-6 is known to modulate the proliferation of different cell types [44,45] and to affect EV release and cargo delivery [46,47], and its metabolites can play a role in EV-mediated cancer invasiveness [48]. Increased proportions of 20:4n-6 and its precursor 20:3n-6 were observed in RA compared to OA synovium [Mustonen et al., unpubl. data]. 20:4n-6 levels in glycero-PLs of bone marrow MSCs have been documented to increase during long-term cultivation at the expense of n-3 PUFAs [49]. These changes could be associated with altered inflammatory signaling and immunomodulatory capacity, and with senescence. Regarding other n-6 PUFAs, 18:2n-6 proportions decreased from control media to FLS-conditioned media, and it was presumably taken up by the cells [50] to promote growth and long-chain n-6 PUFA synthesis. 18:2n-6 is an essential PUFA, the amount of which can be insufficient in culture media [51]. It is capable of influencing EV release [52] and proliferation of different cell types [53,54], and is converted via 18:3n-6 and 20:3n-6 to 20:4n-6, the derivative of which is PGE_2_. PGE_2_ can induce deleterious and beneficial effects on synovial fibroblasts. In RA, it has been demonstrated to both stimulate and inhibit the overgrowth of synovial tissue [24].

FLS proliferation was associated with the up-regulation of *CD44*, *TNF-α*, *PPAR-α*, and *PPAR-γ* expression, while the other measured genes were either down-regulated or did not show significant temporal changes. TNF-*α* is a potent mediator of inflammatory functions that regulate cell death, survival, differentiation, proliferation, and migration [55]. It is overproduced in RA joints and plays a role in the establishment of synovitis, pannus formation, and joint destruction [56]. TNF-*α* is also known to stimulate the biosynthesis of different PL classes by OA FLSs [22]. Based on the present results, we tentatively suggest that, in addition to OA/RA synoviocytes [29], *TNF-α* could participate in the regulation of proliferation of normal FLSs. Moreover, its levels correlated positively with total n-3 and n-6 PUFA percentages and inversely with those of total MUFAs. PPAR-*α* is a transcription factor that functions as a master regulator of hepatic lipid metabolism governing, for instance, FA uptake, binding, and oxidation; ketogenesis; and triacylglycerol (TAG) turnover [57]. In the present study, *PPAR-α* correlated positively with FLS count, but was not associated with FA proportions. FAs and FA-derived compounds, such as eicosanoids, are natural ligands for both PPAR*-α* and PPAR-*γ* [58]. PPAR-*γ* modulates adipogenesis in fat tissues as well as whole-body lipid metabolism and insulin sensitivity. It also induces apoptosis in RA synoviocytes [59] and inhibits the production of inflammatory cytokines in RA and OA synoviocytes [60]. The potential roles of *PPAR-α* and *PPAR-γ* in FLS lipid metabolism remain scarcely investigated. In the present study, *PPAR-γ* was noted to correlate positively with the number of days from passage/confluence and with the proportions of 20:4n-6 and total n-6 PUFAs, while the relationship was negative for total MUFAs.

### 3.3. EV FASs

Bioactive lipids and enzymes involved in their metabolism influence the formation and release of EVs [7,10]. Relatively little is known about the FA profiles of EVs despite their potential role, for instance, in the biosynthesis of LMs. In the present study, 16:0 (31%), 18:1n-9 (25%), 18:0 (20%), 14:0 (6%), and 18:1n-7 (3%) were the individual FAs with the highest proportions in FLS-secreted EVs. These results resemble a previous study where 16:0, 18:0, and 18:1n-9 were the most abundant FAs in EVs from human dermal fibroblasts [23]. In the present experiment, the most abundant individual PUFAs were the essential 18:2n-6 (1.6%) and 18:3n-3 (1.1%), and the percentages of total SFAs, MUFAs, and PUFAs were 61, 32, and 6%, respectively. Compared to parent FLSs, 16:0, 18:0, and 18:1n-9 were among the FAs enriched in the EV fraction (Figure 6A), suggesting selective transfer of FAs from FLSs into EVs. There were no temporal changes in the EV FA profiles during FLS proliferation. However, the proportions of 18:2n-6 in the EV fraction correlated with cell count, which may be associated with the modulation of cell growth, EV release, and LM synthesis [52,54]. Previously, hypoxia induced elevated levels of 18:2n-6 in prostate cancer cell-derived EVs compared to those under normoxic conditions [48].

EVs were previously shown to be enriched with cholesterol, SM, glycosphingolipids, and PS, whereas PC and PI were often more abundant in parent cells [7,8,10,11]. PL species with two saturated fatty acyl groups (e.g., PC 16:0/16:0) can also show increases in EVs. Regarding several lipid classes, species containing 18:0/18:1, 16:0/18:1, and 18:1/18:1 were clearly elevated in EVs [10,11], which supports the present results that showed the enrichment of 16:0, 18:0, and 18:1n-9 in FLS-derived EVs. To the best of our knowledge, this is the first time the FA composition of human FLS EVs has been characterized in detail. This is of importance due to the central role of FLSs in OA and RA and the potential part their EVs play in joint diseases [2]. Previously, dermal fibroblasts were studied in this respect, and their EVs were enriched with lyso-PLs, hydroxylated SM, and ether-linked PLs, while PS and PE reduced compared to parent cells [61]. In addition, total SFA levels increased from cells to EVs [23], similar to the present study. This could lead to increased membrane rigidity and stability of EVs. The present study also observed decreased UFA/SFA ratios in EVs compared to parent FLSs, confirming earlier literature with reduced unsaturation in EVs [7]. Regarding EVs from the metastatic prostate cancer cell line PC-3, fatty acyl chain lengths in PC and PE could be shortened in EXOs [11], also supporting the present results with decreased TACLs in EV total lipids.

The low proportion of PUFAs, such as 20:4n-6, in FLS EVs is intriguing (Figure 6A). The comparison to earlier studies is not always straightforward due to different experimental designs and different ways FA data are expressed. A previous study documented increased amounts of di-PUFA species in specific PL fractions of EVs compared to parent bone marrow MSCs [12]. These cells were treated with PUFA supplements and could potentially have removed excess PUFAs by producing EVs, which may not necessarily represent a natural phenomenon. In dermal fibroblast-derived EVs, the amount of total PUFAs relative to protein content was also increased in respect to parent cells [23]. The lack of enrichment of 24:0 and 24:1n-9 in the present study (Figure 6A) is another finding that calls for explanation. Their proportions were expected to increase due to the importance of sphingolipids in the EV membrane [10], but for instance, in dermal fibroblast-derived EVs, the most common molecular species among SMs was that with 16:0 [61] and, in Sagini et al. [23], 24:1 was below detection. Total DMA proportions were also decreased from FLSs to EVs in the present study, even though plasmalogens can be enriched in EV membranes [62]. However, our FLS EV results show resemblance to EV data from the bronchoalveolar lavage fluid of horses, which also lacked enrichment of PUFAs, 24:0, and 24:1n-9 when EVs were isolated by size-exclusion chromatography [Höglund et al., unpubl. data].

Serum products used for cell cultures contain lipid particles of similar size to EVs [8]. In the present study, fetal bovine serum (FBS) was ultracentrifuged overnight to remove as many EVs and lipid particles as possible before the serum was used on cells. This ultracentrifuged FBS was used until sampling to avoid serum-starvation, which inhibits the proliferation and migration of synoviocytes [63]. Alterations in medium lipid composition would have consequences for the FASs of FLSs and EVs they release [8,10,12], and an abrupt change to serum-free medium could have resulted in reduced EV secretion [31]. We harvested EVs by ultracentrifugation, and this method can result in co-isolation of EVs, lipoproteins, and lipid droplets containing TAGs and cholesteryl esters instead of membrane lipids [10]. The FA analysis of the EV preparation could, thus, have been affected by remnant lipids precipitated by ultracentrifugation. This is known to be a problem especially for plasma samples, but its potential effect cannot be wholly excluded regarding the present results.

In conclusion, the EV release and HA synthesis by FLSs decreased with increasing cell densities. FLS proliferation was featured by elevated 20:4n-6 proportions and reduced n-3/n-6 PUFA ratios, and selective transfer of FAs was documented from FLSs into EVs. While synoviocytes are integral parts of joint anatomy and health, their overgrowth leads to pathological conditions. In the present study, we observed both potentially beneficial (*IL-1β*, *COX-2*, *PPAR-α*, *PPAR-γ*) and detrimental effects (HA, *TNF-α*, 20:4n-6, n-3/n-6 PUFA ratio) of cell growth on the measured variables in normal FLSs. Inflammatory joint diseases, principally OA and RA, display a complex combination of both pro- and anti-inflammatory phenomena, and to influence this balance in a manner beneficial to the patients, basic data on these molecular effects are a prerequisite for the development of novel treatment options. The results offer an attractive starting point for translational studies to enhance the synthesis of molecules beneficial for joint homeostasis and to control those inducing runaway proliferation of FLSs.

## 4. Materials and Methods

### 4.1. Culture and Sampling of FLSs

Normal human FLSs (408-05A; Merck, Darmstadt, Germany) were cultured as monolayers at 37 °C and under 5% CO_2_ in synoviocyte growth medium (415–500; Merck) for passages 3–5/6. Thereafter, the cells were cultured in synoviocyte growth medium (415F-500; Merck) supplemented with 10% FBS (HyClone Laboratories, Logan, UT, USA) and, at passage 7/8, with 10% EV-free FBS. To obtain this, the HyClone FBS product was ultracentrifuged at 100,000× *g* for 16 h [64] in order to minimize the presence of EVs and lipoproteins [10], followed by sterile filtering (pore size 0.22 μm; Sartorius, Göttingen, Germany). The medium was replaced every 48–72 h.

The cells were first grown in T25 tissue culture flasks with 5 mL (≤60% confluence) or 7 mL of medium (>60% confluence). Once they reached 80% confluence, they were transferred into T75 culture flasks with 15 mL (≤60% confluence) or 20–25 mL (>60% confluence) of medium. Sampling was conducted on 6 cm culture dishes (21 cm^2^) after passage 7/8 with 5 mL (≤60% confluence) or 7 mL of medium (>60% confluence). The cells and media were harvested at timepoints 1 (24 h from passage), 2 (9–12 days from passage, 60% confluence), 3 (11–17 days from passage, 80% confluence), 4 (18–24 days from passage, 7 days from timepoint 3), and 5 (25–31 days from passage, 7 days from timepoint 4). Timepoint 1 is the lag phase with minimal cell growth, timepoints 2–3 depict the proliferation phase, and timepoints 4–5 represent high-density cells with reduced proliferation.

The media from the culture flasks were sterile-filtered (5 μm; Sartorius) and stored as aliquots at −80 °C. The cells were washed with phosphate buffered saline (PBS; Corning, Manassas, VA, USA), detached with Trypsin-EDTA solution (T3924; Merck), followed by a treatment with trypsin inhibitor (T6414; Merck) and centrifugation at 220× *g* for 5 min. The pellet was diluted in sterile-filtered PBS (0.22 μm) and the total cell count was estimated. The cells were divided into two Eppendorf tubes and centrifuged at 2300× *g* for 5 min. One of the precipitates was diluted in 100 µL of sterile-filtered PBS for FA analysis and the other in 200 µL of TRI Reagent (T9424; Merck) for RNA isolation, and both were stored at −80 °C. Altogether, 9 media and cell samples were obtained per timepoint, but all analyses could not be conducted on every sample due to their limited volume.

### 4.2. FA Determination

Harvested sterile-filtered media (300 μL) and FLSs were transmethylated in methanolic H_2_SO_4_ under nitrogen atmosphere, and the formed FA methyl esters were extracted with hexane and analyzed by the Shimadzu GC-2010 Plus gas chromatograph (Shimadzu, Kyoto, Japan) [65,66]. Another subsample of sterile-filtered media (3 mL) was centrifuged at 1000× *g* for 10 min at 4 °C, and the supernatant at 1200× *g* for 20 min at 4 °C. Finally, the supernatant was ultracentrifuged at <110,000× *g* for 90 min at 4 °C, and the EV pellet which would be equivalent to the EV secretion by an average of 85,000 cells for 2 days was diluted in sterile-filtered PBS and analyzed with gas chromatography. The FA methyl ester structures were confirmed by using electron impact mass spectra recorded by the Shimadzu GCMS-QP2010 Ultra with the mass selective detector. The results represent the FA composition (mol-%) of total lipids in the media, cells, or EV fraction. The DBI and TACL were calculated as previously described [65].

### 4.3. qPCR of FLSs

Total cellular RNA was extracted using TRI Reagent combined with standard chloroform–isopropanol precipitation. RNA concentration was measured with the NanoDrop ONE (Thermo Fisher Scientific, Waltham, MA, USA), and 1000 ng was synthesized to cDNA using Verso cDNA Synthesis Kit (Thermo Fisher Scientific) in the Biometra Personal Cycler (Analytik Jena GmbH, Jena, Germany). Eight biological replicates were analyzed using LightCycler 480 SYBR Green I Master reagent and the LightCycler 480 polymerase chain reaction apparatus (Roche, Basel, Switzerland) under the following thermal conditions: 95 °C for 5 min, 45 cycles of 95 °C for 20 s, 60 °C for 20 s, and 72 °C for 20 s, followed by 72 °C for 5 min. Melting curve analysis was performed in the range of 40 to 95 °C, 0.5 °C for each 2-s interval. Ribosomal protein lateral stalk subunit P0 (*RPLP0*) was used to normalize the amounts of mRNA between samples to get the relative mRNA levels at each timepoint. Fold changes were calculated using the formula 2-ΔΔCt, where ΔΔCt is the ΔCt_(differentiated)_ − ΔCt_(non-differentiated)_, where ΔCt is Ct_(target gene)_ − Ct _(*RPLP0*)_, and Ct is the cycle at which the threshold is crossed. The relative gene expression was set to 1 for timepoint 1 samples. Primer sequences are reported in Table S2.

### 4.4. CLSM

Eight-well ibidi chambers (ibidi GmbH, Gräfelfing, Germany) were coated with 10 µg/mL poly-D-lysine hydrobromide (P6407; Merck) as previously described [67]. The ultracentrifuged medium-EV samples were incubated overnight in eight-well chambers, followed by staining with CellMask Deep Red plasma membrane stain (Life Technologies, Eugene, OR, USA), Alexa Fluor 568-labeled HABC [68], Alexa Fluor 488-labeled CD63 antibody (BioLegend, San Diego, CA, USA), and/or Alexa Fluor 594-labeled phalloidin-iFluor (Abcam, Cambridge, UK). CD63 and phalloidin are established markers of transmembrane and cytosolic proteins, respectively, recovered in EVs [25]. The specificity of the stainings was validated by samples containing only medium-EVs and buffer and probes and buffer, respectively. For the quantitative measurement of EVs, HA-particles, and HA–EVs, sterile-filtered but otherwise unprocessed conditioned media were stained with CellMask Deep Red plasma membrane stain and HABC by using poly-D-lysine hydrobromide-coated ibidi chambers. FLSs were stained with CellMask Deep Red plasma membrane stain, HABC, anti-CD44 monoclonal antibody (MRQ-13; Merck), and NucBlue Live Cell Stain ReadyProbes reagent (Life Technologies).

CLSM was performed with the Zeiss Axio Observer inverted microscope equipped with the Zeiss LSM 800 confocal module (Carl Zeiss MicroImaging GmbH, Jena). Image acquisition was carried out using the ZEN 2.3 blue edition software (Carl Zeiss MicroImaging GmbH) as previously outlined [67]. The area and intensity of the stainings, counts of EVs, HA-particles, and HA–EVs, and size distribution of EVs and HA-particles were determined with the ImageJ/Fiji *v*1.53 software (NIH, Bethesda, MA) with various open-source plug-ins. Co-localization of EV and HA fluorescences was determined with the ComDet analysis (*v*0.4.2).

### 4.5. NTA

The sterile-filtered FLS-conditioned media were first ultracentrifuged at 110,000× *g* for 2 h and again for 90 min to wash the pellet, followed by resuspension in 100 µL of PBS. These enriched EV samples were analyzed by NTA using the Nanosight model LM14 (Malvern Panalytical, Malvern, UK) equipped with a blue laser (404 nm, 70 mW) and sCMOS camera (Hamamatsu Photonics, Hamamatsu, Japan). The samples were diluted in 0.1 µm-filtered (Millex-VV; Merck) PBS to obtain 40–100 particles/view, and five 30 s videos were recorded using camera level 14. The data were analyzed using the NTA software *v*3.0 (Malvern Panalytical) with the detection threshold 5 to track as many particles as possible with minimal background.

### 4.6. HA Determination

Temporal changes in the HA concentrations of FLS-conditioned medium were determined with a sandwich-type enzyme-linked sorbent assay [69].

### 4.7. Statistical Analyses

All statistical analyses were conducted with the IBM SPSS *v*25 software (IBM, Armonk, NY, USA). Comparisons between the sample types or timepoints of 9 experiments were performed with the Kruskal–Wallis ANOVA. Correlations were calculated with the Spearman correlation coefficient (r_s_). The *p* value < 0.05 was considered statistically significant. The results are presented as the mean ± SE. To analyze how clearly the different sample types and timepoints differed from one another and which variables separated them most clearly, we also performed the discriminant analysis for the FA data.

## Figures and Tables

**Table 1:** Confocal microscopy data of EVs released by fibroblast-like synoviocytes into conditioned media at timepoints 1–5 (mean ± SE).

Timepoint	1	2	3	4	5	*p*
Area of EVs, µm^2^	1.74 ± 0.063 ^a^	1.86 ± 0.285 ^ab^	2.32 ± 0.170 ^bc^	2.46 ± 0.448 ^bc^	2.63 ± 0.278 ^c^	0.039
Intensity of EVs, AU	25.46 ± 1.716	20.62 ± 1.814	23.73 ± 3.408	25.12 ± 2.996	23.24 ± 2.264	0.583
Count of EVs, *n*	28 ± 5 ^b^	31 ± 7 ^b^	18 ± 1 ^ab^	18 ± 2 ^ab^	16 ± 1 ^a^	0.033
Diameter of EVs, µm	1.41 ± 0.024 ^a^	1.38 ± 0.115 ^ab^	1.60 ± 0.061 ^bc^	1.63 ± 0.162 ^abc^	1.68 ± 0.094 ^c^	0.041
Area of HA-particles, µm^2^	0.63 ± 0.065	0.77 ± 0.112	0.78 ± 0.114	0.86 ± 0.171	0.91 ± 0.176	0.614
Intensity of HA-particles, AU	96.21 ± 4.935	100.29 ± 3.932	89.90 ± 7.251	95.39 ± 4.083	96.95 ± 4.451	0.703
Count of HA-particles, *n*	23 ± 5	13 ± 2	12 ± 1	13 ± 1	10 ± 1	0.051
Diameter of HA-particles, μm	0.85 ± 0.041	1.02 ± 0.136	0.92 ± 0.060	0.96 ± 0.092	1.14 ± 0.266	0.713
Area of HA–EVs, µm^2^	0.86 ± 0.042	0.96 ± 0.077	1.02 ± 0.083	1.05 ± 0.111	1.19 ± 0.153	0.243
Count of HA–EVs, *n*	23 ± 4 ^b^	12 ± 1 ^a^	13 ± 0.4 ^ab^	14 ± 2 ^ab^	11 ± 1 ^a^	0.042
Diameter of HA–EVs, μm	1.04 ± 0.025	1.10 ± 0.045	1.13 ± 0.045	1.15 ± 0.059	1.22 ± 0.077	0.243
Co-localization of EVs and HA, %	84 ± 7	49 ± 9	75 ± 6	83 ± 12	74 ± 7	0.078

EV = extracellular vesicle, AU = arbitrary unit, HA = hyaluronan, HA–EV = HA-containing EV; 1 = lag phase, 2–3 = proliferating cells, 4–5 = high-density cells; means with dissimilar superscript letters indicate significant differences between timepoints within a row (Kruskal–Wallis ANOVA).

## Data Availability

All relevant data analyzed during this study are included in this published article and its supplementary information files.

## Supplementary Materials

The following supporting information can be downloaded at: https://www.mdpi.com/article/10.3390/ijms23105613/s1.

## List of Abbreviations

AB = apoptotic body, ANOVA = analysis of variance, CD = cluster of differentiation, CLSM = confocal laser scanning microscopy, COX = cyclooxygenase, DBI = double bond index, DMA = dimethyl acetal, i.e., plasmalogen alkenyl chain derivative, EV = extracellular vesicle, EXO = exosome, FA = fatty acid, FAS = fatty acid signature, FBS = fetal bovine serum, FLS = fibroblast-like synoviocyte, HA = hyaluronan, HABC = hyaluronan binding complex, HA–EV = hyaluronan-containing extracellular vesicle, HAS = hyaluronan synthase, IL = interleukin, LM = lipid mediator, MSC = mesenchymal stem (stromal) cell, MUFA = monounsaturated fatty acid, MV = microvesicle, NTA = nanoparticle tracking analysis, OA = osteoarthritis, PBS = phosphate buffered saline, PC = phosphatidylcholine, PE = phosphatidylethanolamine, PGE_2_ = prostaglandin E_2_, PI = phosphatidylinositol, PL = phospholipid, PPAR = peroxisome proliferator-activated receptor, PS = phosphatidylserine, PUFA = polyunsaturated fatty acid, qPCR = quantitative polymerase chain reaction, RA = rheumatoid arthritis, RPLP0 = ribosomal protein lateral stalk subunit P0, r_s_ = Spearman correlation coefficient, SF = synovial fluid, SFA = saturated fatty acid, SM = sphingomyelin, TACL = total average chain length, TAG = triacylglycerol, TNF = tumor necrosis factor, UFA = unsaturated fatty acid.

## References

[1] Rilla K., Mustonen A.-M., Arasu U.T., Härkönen K., Matilainen J., Nieminen P. (2019). Extracellular vesicles are integral and functional components of the extracellular matrix. Matrix Biol..

[2] Mustonen A.-M., Nieminen P. (2021). Extracellular vesicles and their potential significance in the pathogenesis and treatment of osteoarthritis. Pharmaceuticals.

[3] Mustonen A.-M., Nieminen P., Joukainen A., Jaroma A., Kääriäinen T., Kröger H., Lázaro-Ibáñez E., Siljander P.R.-M., Kärjä V., Härkönen K. (2016). First in vivo detection and characterization of hyaluronan-coated extracellular vesicles in human synovial fluid. J. Orthop. Res..

[4] Kosinska M.K., Ludwig T.E., Liebisch G., Zhang R., Siebert H.-C., Wilhelm J., Kaesser U., Dettmeyer R.B., Klein H., Ishaque B. (2015). Articular joint lubricants during osteoarthritis and rheumatoid arthritis display altered levels and molecular species. PLoS ONE.

[5] Altman R.D., Manjoo A., Fierlinger A., Niazi F., Nicholls M. (2015). The mechanism of action for hyaluronic acid treatment in the osteoarthritic knee: A systematic review. BMC Musculoskelet. Disord..

[6] Ragni E., Perucca Orfei C., De Luca P., Lugano G., Viganò M., Colombini A., Valli F., Zacchetti D., Bollati V., de Girolamo L. (2019). Interaction with hyaluronan matrix and miRNA cargo as contributors for in vitro potential of mesenchymal stem cell-derived extracellular vesicles in a model of human osteoarthritic synoviocytes. Stem. Cell Res. Ther..

[7] Skotland T., Hessvik N.P., Sandvig K., Llorente A. (2019). Exosomal lipid composition and the role of ether lipids and phosphoinositides in exosome biology. J. Lipid Res..

[8] Skotland T., Sagini K., Sandvig K., Llorente A. (2020). An emerging focus on lipids in extracellular vesicles. Adv. Drug Deliv. Rev..

[9] Maeda-Sano K., Gotoh M., Morohoshi T., Someya T., Murofushi H., Murakami-Murofushi K. (2014). Cyclic phosphatidic acid and lysophosphatidic acid induce hyaluronic acid synthesis via CREB transcription factor regulation in human skin fibroblasts. Biochim. Et Biophys. Acta.

[10] Skotland T., Sandvig K., Llorente A. (2017). Lipids in exosomes: Current knowledge and the way forward. Prog. Lipid Res..

[11] Llorente A., Skotland T., Sylvänne T., Kauhanen D., Róg T., Orłowski A., Vattulainen I., Ekroos K., Sandvig K. (2013). Molecular lipidomics of exosomes released by PC-3 prostate cancer cells. Biochim. Et Biophys. Acta.

[12] Holopainen M., Colas R.A., Valkonen S., Tigistu-Sahle F., Hyvärinen K., Mazzacuva F., Lehenkari P., Käkelä R., Dalli J., Kerkelä E. (2019). Polyunsaturated fatty acids modify the extracellular vesicle membranes and increase the production of proresolving lipid mediators of human mesenchymal stromal cells. Biochim. Et Biophys. Acta (BBA)-Mol. Cell Biol. Lipids.

[13] Record M., Carayon K., Poirot M., Silvente-Poirot S. (2014). Exosomes as new vesicular lipid transporters involved in cell–cell communication and various pathophysiologies. Biochim. Et Biophys. Acta.

[14] Valkonen S., Holopainen M., Colas R.A., Impola U., Dalli J., Käkelä R., Siljander P.R.-M., Laitinen S. (2019). Lipid mediators in platelet concentrate and extracellular vesicles: Molecular mechanisms from membrane glycerophospholipids to bioactive molecules. Biochim. Et Biophys. Acta (BBA)-Mol. Cell Biol. Lipids.

[15] Bustamante M.F., Garcia-Carbonell R., Whisenant K.D., Guma M. (2017). Fibroblast-like synoviocyte metabolism in the pathogenesis of rheumatoid arthritis. Arthritis Res. Ther..

[16] Han D., Fang Y., Tan X., Jiang H., Gong X., Wang X., Hong W., Tu J., Wei W. (2020). The emerging role of fibroblast-like synoviocytes-mediated synovitis in osteoarthritis: An update. J. Cell. Mol. Med..

[17] Distler J.H.W., Jüngel A., Huber L.C., Seemayer C.A., Reich C.F., Gay R.E., Michel B.A., Fontana A., Gay S., Pisetsky D.S. (2005). The induction of matrix metalloproteinase and cytokine expression in synovial fibroblasts stimulated with immune cell microparticles. Proc. Natl. Acad. Sci. USA.

[18] Berckmans R.J., Nieuwland R., Kraan M.C., Schaap M.C.L., Pots D., Smeets T.J.M., Sturk A., Tak P.P. (2005). Synovial microparticles from arthritic patients modulate chemokine and cytokine release by synoviocytes. Arthritis Res. Ther..

[19] Boilard E., Nigrovic P.A., Larabee K., Watts G.F.M., Coblyn J.S., Weinblatt M.E., Massarotti E.M., Remold-O’Donnell E., Farndale R.W., Ware J. (2010). Platelets amplify inflammation in arthritis via collagen-dependent microparticle production. Science.

[20] Kato T., Miyaki S., Ishitobi H., Nakamura Y., Nakasa T., Lotz M.K., Ochi M. (2014). Exosomes from IL-1β stimulated synovial fibroblasts induce osteoarthritic changes in articular chondrocytes. Arthritis Res. Ther..

[21] David-Raoudi M., Deschrevel B., Leclercq S., Galéra P., Boumediene K., Pujol J.-P. (2009). Chondroitin sulfate increases hyaluronan production by human synoviocytes through differential regulation of hyaluronan synthases: Role of p38 and Akt. Arthritis Rheum..

[22] Sluzalska K.D., Liebisch G., Lochnit G., Ishaque B., Hackstein H., Schmitz G., Rickert M., Steinmeyer J. (2017). Interleukin-1β affects the phospholipid biosynthesis of fibroblast-like synoviocytes from human osteoarthritic knee joints. Osteoarthr. Cartil..

[23] Sagini K., Urbanelli L., Costanzi E., Mitro N., Caruso D., Emiliani C., Buratta S. (2018). Oncogenic H-Ras expression induces fatty acid profile changes in human fibroblasts and extracellular vesicles. Int. J. Mol. Sci..

[24] Mustonen A.-M., Nieminen P. (2021). Fatty acids and oxylipins in osteoarthritis and rheumatoid arthritis—A complex field with significant potential for future treatments. Curr. Rheumatol. Rep..

[25] Théry C., Witwer K.W., Aikawa E., Alcaraz M.J., Anderson J.D., Andriantsitohaina R., Antoniou A., Arab T., Archer F., Atkin-Smith G.K. (2018). Minimal information for studies of extracellular vesicles 2018 (MISEV2018): A position statement of the International Society for Extracellular Vesicles and update of the MISEV2014 guidelines. J. Extracell. Vesicles.

[26] Recklies A.D., White C., Melching L., Roughley P.J. (2001). Differential regulation and expression of hyaluronan synthases in human articular chondrocytes, synovial cells and osteosarcoma cells. Biochem. J..

[27] Itano N., Sawai T., Yoshida M., Lenas P., Yamada Y., Imagawa M., Shinomura T., Hamaguchi M., Yoshida Y., Ohnuki Y. (1999). Three isoforms of mammalian hyaluronan synthases have distinct enzymatic properties. J. Biol. Chem..

[28] Hronowski L., Anastassiades T.P. (1980). The effect of cell density on net rates of glycosaminoglycan synthesis and secretion by cultured rat fibroblasts. J. Biol. Chem..

[29] Blewis M.E., Lao B.J., Schumacher B.L., Bugbee W.D., Sah R.L., Firestein G.S. (2010). Interactive cytokine regulation of synoviocyte lubricant secretion. Tissue Eng. Part A.

[30] Goldberg R.L., Toole B.P. (1987). Hyaluronate inhibition of cell proliferation. Arthritis Rheum..

[31] Gudbergsson J.M., Johnsen K.B., Skov M.N., Duroux M. (2016). Systematic review of factors influencing extracellular vesicle yield from cell cultures. Cytotechnology.

[32] Patel D.B., Gray K.M., Santharam Y., Lamichhane T.N., Stroka K.M., Jay S.M. (2017). Impact of cell culture parameters on production and vascularization bioactivity of mesenchymal stem cell-derived extracellular vesicles. Bioeng. Transl. Med..

[33] Jimenez L., Yu H., McKenzie A.J., Franklin J.L., Patton J.G., Liu Q., Weaver A.M. (2019). Quantitative proteomic analysis of small and large extracellular vesicles (EVs) reveals enrichment of adhesion proteins in small EVs. J. Proteome Res..

[34] Wang R., Jiang W., Zhang L., Xie S., Zhang S., Yuan S., Jin Y., Zhou G. (2020). Intra-articular delivery of extracellular vesicles secreted by chondrogenic progenitor cells from MRL/MpJ superhealer mice enhances articular cartilage repair in a mouse injury model. Stem Cell Res. Ther..

[35] Sun X., Zhu H., Li W., Zhao L., Li W., Li X., Xie Z. (2021). Small extracellular vesicles secreted by vaginal fibroblasts exert inhibitory effect in female stress urinary incontinence through regulating the function of fibroblasts. PLoS ONE.

[36] Tan F., Wang D., Yuan Z. (2020). The fibroblast-like synoviocyte derived exosomal long non-coding RNA H19 alleviates osteoarthritis progression through the miR-106b-5p/TIMP2 axis. Inflammation.

[37] Wu H., Zhou X., Wang X., Cheng W., Hu X., Wang Y., Luo B., Huang W., Gu J. (2021). MiR-34a in extracellular vesicles from bone marrow mesenchymal stem cells reduces rheumatoid arthritis inflammation via the cyclin I/ATM/ATR/p53 axis. J. Cell. Mol. Med..

[38] Neumann E., Riepl B., Knedla A., Lefèvre S., Tarner I.H., Grifka J., Steinmeyer J., Schölmerich J., Gay S., Müller-Ladner U. (2010). Cell culture and passaging alters gene expression pattern and proliferation rate in rheumatoid arthritis synovial fibroblasts. Arthritis Res. Ther..

[39] Kavaliauskiene S., Nymark C.-M., Bergan J., Simm R., Sylvänne T., Simolin H., Ekroos K., Skotland T., Sandvig K. (2014). Cell density-induced changes in lipid composition and intracellular trafficking. Cell. Mol. Life Sci..

[40] Munir R., Lisec J., Swinnen J.V., Zaidi N. (2019). Lipid metabolism in cancer cells under metabolic stress. Br. J. Cancer.

[41] Hall Z., Chiarugi D., Charidemou E., Leslie J., Scott E., Pellegrinet L., Allison M., Mocciaro G., Anstee Q.M., Evan G.I. (2021). Lipid remodeling in hepatocyte proliferation and hepatocellular carcinoma. Hepatology.

[42] Connolly K.D., Guschina I.A., Yeung V., Clayton A., Draman M.S., Von Ruhland C., Ludgate M., James P.E., Rees D.A. (2015). Characterisation of adipocyte-derived extracellular vesicles released pre- and post-adipogenesis. J. Extracell. Vesicles.

[43] Spector A.A., Yorek M.A. (1985). Membrane lipid composition and cellular function. J. Lipid Res..

[44] Spector A.A., Kiser R.E., Denning G.M., Koh S.-W.M., DeBault L.E. (1979). Modification of the fatty acid composition of cultured human fibroblasts. J. Lipid Res..

[45] Leng X., Jiang H. (2019). Effects of arachidonic acid and its major prostaglandin derivatives on bovine myoblast proliferation, differentiation, and fusion. Domest. Anim. Endocrinol..

[46] Ponomareva A.A., Nevzorova T.A., Mordakhanova E.R., Andrianova I.A., Rauova L., Litvinov R.I., Weisel J.W. (2017). Intracellular origin and ultrastructure of platelet-derived microparticles. J. Thromb. Haemost..

[47] Gasperi V., Vangapandu C., Savini I., Ventimiglia G., Adorno G., Catani M.V. (2019). Polyunsaturated fatty acids modulate the delivery of platelet microvesicle-derived microRNAs into human breast cancer cell lines. J. Nutr. Biochem..

[48] Schlaepfer I.R., Nambiar D.K., Ramteke A., Kumar R., Dhar D., Agarwal C., Bergman B., Graner M., Maroni P., Singh R.P. (2015). Hypoxia induces triglycerides accumulation in prostate cancer cells and extracellular vesicles supporting growth and invasiveness following reoxygenation. Oncotarget.

[49] Kilpinen L., Tigistu-Sahle F., Oja S., Greco D., Parmar A., Saavalainen P., Nikkilä J., Korhonen M., Lehenkari P., Käkelä R. (2013). Aging bone marrow mesenchymal stromal cells have altered membrane glycerophospholipid composition and functionality. J. Lipid Res..

[50] Bettger W.J., Driscoll E.R., Karmiol S. (1992). Selective depletion of non-esterified fatty acids in fetal bovine serum-supplemented culture medium by human fibroblasts proliferating in low-density culture. J. Nutr. Biochem..

[51] Ouellette M.-È., Bérubé J.-C., Bourget J.-M., Vallée M., Bossé Y., Fradette J. (2019). Linoleic acid supplementation of cell culture media influences the phospholipid and lipid profiles of human reconstructed adipose tissue. PLoS ONE.

[52] Garcia-Hernandez A., Leal-Orta E., Ramirez-Ricardo J., Cortes-Reynosa P., Thompson-Bonilla R., Salazar E.P. (2021). Linoleic acid induces secretion of extracellular vesicles from MDA-MB-231 breast cancer cells that mediate cellular processes involved with angiogenesis in HUVECs. Prostaglandins Other Lipid Mediat..

[53] Meng H., Shen Y., Shen J., Zhou F., Shen S., Das U.N. (2013). Effect of n-3 and n-6 unsaturated fatty acids on prostate cancer (PC-3) and prostate epithelial (RWPE-1) cells in vitro. Lipids Health Dis..

[54] Mouradian M., Kikawa K.D., Johnson E.D., Beck K.L., Pardini R.S. (2014). Key roles for GRB2-associated-binding protein 1, phosphatidylinositol-3-kinase, cyclooxygenase 2, prostaglandin E2 and transforming growth factor alpha in linoleic acid-induced upregulation of lung and breast cancer cell growth. Prostaglandins Leukot. Essent. Fatty Acids.

[55] Bradley J.R. (2008). TNF-mediated inflammatory disease. J. Pathol..

[56] Camussi G., Lupia E. (1998). The future role of anti-tumour necrosis factor (TNF) products in the treatment of rheumatoid arthritis. Drugs.

[57] Kersten S. (2014). Integrated physiology and systems biology of PPARα. Mol. Metab..

[58] Ahmadian M., Suh J.M., Hah N., Liddle C., Atkins A.R., Downes M., Evans R.M. (2013). PPARγ signaling and metabolism: The good, the bad and the future. Nat. Med..

[59] Kawahito Y., Kondo M., Tsubouchi Y., Hashiramoto A., Bishop-Bailey D., Inoue K.-i., Kohno M., Yamada R., Hla T., Sano H. (2000). 15-Deoxy-∆^12,14^-PGJ_2_ induces synoviocyte apoptosis and suppresses adjuvant-induced arthritis in rats. J. Clin. Investig..

[60] Ji J.D., Cheon H., Jun J.B., Choi S.J., Kim Y.R., Lee Y.H., Kim T.H., Chae I.J., Song G.G., Yoo D.H. (2001). Effects of peroxisome proliferator-activated receptor-γ (PPAR-γ) on the expression of inflammatory cytokines and apoptosis induction in rheumatoid synovial fibroblasts and monocytes. J. Autoimmun..

[61] Buratta S., Urbanelli L., Sagini K., Giovagnoli S., Caponi S., Fioretto D., Mitro N., Caruso D., Emiliani C. (2017). Extracellular vesicles released by fibroblasts undergoing H-Ras induced senescence show changes in lipid profile. PLoS ONE.

[62] Lydic T.A., Townsend S., Adda C.G., Collins C., Mathivanan S., Reid G.E. (2015). Rapid and comprehensive ‘shotgun’ lipidome profiling of colorectal cancer cell derived exosomes. Methods.

[63] Amrichová J., Špaková T., Rosocha J., Harvanová D., Bačenková D., Lacko M., Horňák S. (2014). Effect of PRP and PPP on proliferation and migration of human chondrocytes and synoviocytes in vitro. Cent. Eur. J. Biol..

[64] Shelke G.V., Lässer C., Gho Y.S., Lötvall J. (2014). Importance of exosome depletion protocols to eliminate functional and RNA-containing extracellular vesicles from fetal bovine serum. J. Extracell. Vesicles.

[65] Mustonen A.-M., Käkelä R., Lehenkari P., Huhtakangas J., Turunen S., Joukainen A., Kääriäinen T., Paakkonen T., Kröger H., Nieminen P. (2019). Distinct fatty acid signatures in infrapatellar fat pad and synovial fluid of patients with osteoarthritis versus rheumatoid arthritis. Arthritis Res. Ther..

[66] Mustonen A.-M., Käkelä R., Joukainen A., Lehenkari P., Jaroma A., Kääriäinen T., Kröger H., Paakkonen T., Sihvo S.P., Nieminen P. (2021). Synovial fluid fatty acid profiles are differently altered by inflammatory joint pathologies in the shoulder and knee joints. Biology.

[67] Mustonen A.-M., Capra J., Rilla K., Lehenkari P., Oikari S., Kääriäinen T., Joukainen A., Kröger H., Paakkonen T., Matilainen J. (2021). Characterization of hyaluronan-coated extracellular vesicles in synovial fluid of patients with osteoarthritis and rheumatoid arthritis. BMC Musculoskelet. Disord..

[68] Rilla K., Tiihonen R., Kultti A., Tammi M., Tammi R. (2008). Pericellular hyaluronan coat visualized in live cells with a fluorescent probe is scaffolded by plasma membrane protrusions. J. Histochem. Cytochem..

[69] Hiltunen E.L.J., Anttila M., Kultti A., Ropponen K., Penttinen J., Yliskoski M., Kuronen A.T., Juhola M., Tammi R., Tammi M. (2002). Elevated hyaluronan concentration without hyaluronidase activation in malignant epithelial ovarian tumors. Cancer Res..

