# Supplementary material for: Fatty Acid Fingerprints and Hyaluronic Acid in Extracellular Vesicles from Proliferating Human Fibroblast-like Synoviocytes
Source: Int J Mol Sci. 2022 May 17;23(10):5613. doi: 10.3390/ijms23105613 (PMC9146557; doi:10.3390/ijms23105613)
Supplement: Supplementary file 1 [file ijms-23-05613-s001.zip › ijms-1669823-supplementary.pdf]

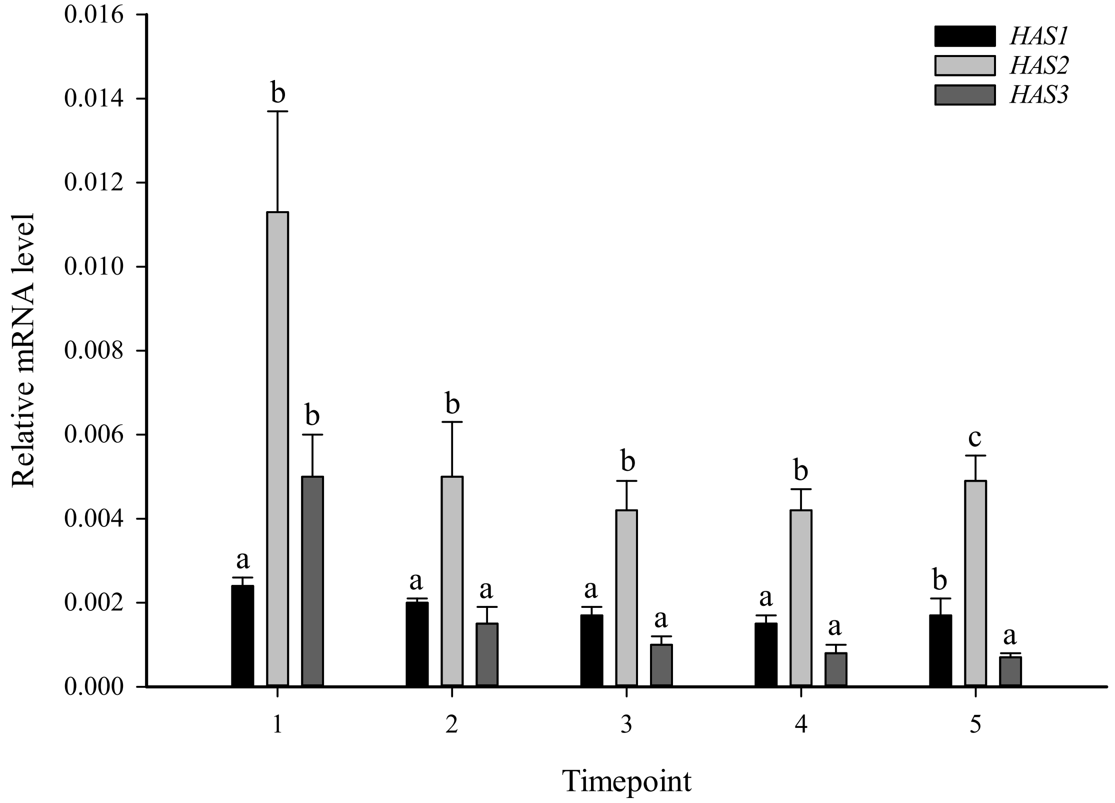

Figure S1. Expression profiling of the human hyaluronan synthase (*HAS*) gene family in fibroblast-like synoviocytes (mean + SE) at timepoint 1 = lag phase, timepoints 2–3 = proliferating cells, and timepoints 4–5 = high-density cells. Real-time quantitative PCR was used to determine the ratio of the mRNA expression of the three *HAS* genes relative to the control gene ribosomal protein lateral stalk subunit P0. Means with dissimilar letters indicate significant differences between the isoforms within a timepoint (Kruskal–Wallis ANOVA, $p < 0.05$).

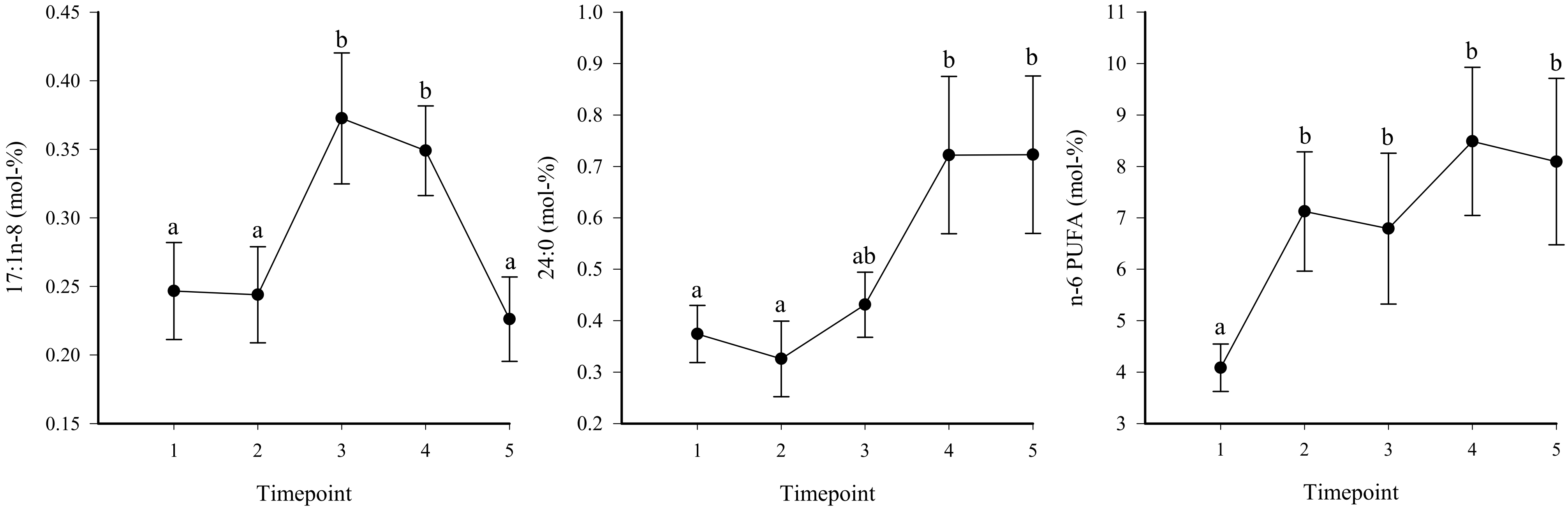

Figure S2. Temporal changes in the proportions of 17:1n-8, 24:0, and the sum of n-6 polyunsaturated fatty acids (PUFA) in fibroblast-like synoviocytes. Timepoint 1 = lag phase, timepoints 2–3 = proliferating cells, timepoints 4–5 = high-density cells. Dissimilar letters above means ± SE indicate significant differences between timepoints (Kruskal–Wallis ANOVA, $p < 0.05$).

Table S1. Fatty acid composition of the media, cells, and ultracentrifuged fraction (mean ± SE).

Fatty acid	Control medium	Conditioned medium	Fibroblast-like synoviocytes	Extracellular vesicles	*p*
14:0	4.718 ± 0.168^{a}	4.678 ± 0.094^{a}	5.258 ± 0.166^{b}	5.618 ± 0.103^{b}	<0.0005
14:1n-5	0.399 ± 0.040^{b}	0.293 ± 0.017^{a}	0.434 ± 0.028^{b}	0.444 ± 0.024^{b}	<0.0005
15:0*i*	0.262 ± 0.023^{bc}	0.220 ± 0.013^{b}	0.291 ± 0.018^{c}	0.153 ± 0.010^{a}	<0.0005
15:0*ai*	0.378 ± 0.034^{a}	0.364 ± 0.018^{a}	0.563 ± 0.033^{b}	0.428 ± 0.023^{a}	<0.0005
15:0	0.288 ± 0.031^{b}	0.268 ± 0.013^{b}	0.284 ± 0.018^{b}	0.205 ± 0.014^{a}	<0.0005
DMA 16:0	0.651 ± 0.071^{a}	0.662 ± 0.055^{a}	1.190 ± 0.072^{b}	0.779 ± 0.055^{a}	<0.0005
16:0	30.563 ± 0.618^{b}	29.849 ± 0.466^{b}	25.963 ± 0.446^{a}	30.758 ± 0.298^{b}	<0.0005
16:1n-9	0.311 ± 0.035^{a}	0.466 ± 0.031^{b}	0.612 ± 0.033^{c}	0.353 ± 0.022^{a}	<0.0005
16:1n-7	1.309 ± 0.059	1.392 ± 0.046	1.479 ± 0.046	1.413 ± 0.043	0.125
16:1n-5	0.248 ± 0.032^{a}	0.349 ± 0.026^{ab}	0.501 ± 0.035^{c}	0.388 ± 0.032^{b}	0.001
17:0*i*	1.065 ± 0.060^{a}	1.005 ± 0.057^{a}	1.306 ± 0.065^{b}	1.096 ± 0.042^{a}	0.004
17:0*ai*	0.360 ± 0.048	0.281 ± 0.014	0.321 ± 0.017	0.307 ± 0.016	0.201
17:0	0.923 ± 0.025^{c}	0.850 ± 0.022^{bc}	0.817 ± 0.032^{b}	0.735 ± 0.020^{a}	<0.0005
17:1n-8	0.339 ± 0.042^{b}	0.309 ± 0.019^{b}	0.289 ± 0.018^{ab}	0.240 ± 0.015^{a}	0.025
DMA 18:0	0.558 ± 0.058^{a}	0.521 ± 0.035^{a}	1.009 ± 0.057^{b}	0.612 ± 0.030^{a}	<0.0005
18:0	19.297 ± 0.615^{ab}	20.478 ± 0.326^{b}	18.605 ± 0.233^{a}	19.886 ± 0.144^{b}	<0.0005
18:1n-9	24.562 ± 0.252^{bc}	23.703 ± 0.289^{b}	22.264 ± 0.400^{a}	24.640 ± 0.223^{c}	<0.0005
18:1n-7	3.578 ± 0.203^{b}	3.443 ± 0.111^{b}	3.539 ± 0.076^{b}	2.850 ± 0.069^{a}	<0.0005
18:1n-5	1.428 ± 0.152^{ab}	1.433 ± 0.071^{a}	1.740 ± 0.082^{b}	1.718 ± 0.076^{b}	0.009
18:2n-6	1.875 ± 0.069^{c}	1.693 ± 0.040^{b}	1.623 ± 0.032^{ab}	1.578 ± 0.026^{a}	0.001
18:3n-6	0.358 ± 0.043^{ab}	0.416 ± 0.049^{a}	0.556 ± 0.036^{c}	0.604 ± 0.079^{bc}	0.002
18:3n-3	0.675 ± 0.056^{a}	0.711 ± 0.053^{a}	0.989 ± 0.054^{b}	1.063 ± 0.060^{b}	<0.0005
20:0	0.374 ± 0.045	0.448 ± 0.029	0.474 ± 0.029	0.385 ± 0.017	0.178
20:1n-9	0.196 ± 0.017	0.235 ± 0.021	0.270 ± 0.018	0.219 ± 0.012	0.100
20:3n-6	0.675 ± 0.117^{ab}	0.742 ± 0.070^{b}	1.184 ± 0.072^{c}	0.453 ± 0.033^{a}	<0.0005
20:4n-6	0.872 ± 0.200^{b}	1.086 ± 0.107^{b}	3.294 ± 0.543^{c}	0.355 ± 0.023^{a}	<0.0005
20:5n-3	0.394 ± 0.064^{b}	0.396 ± 0.050^{b}	0.435 ± 0.040^{b}	0.227 ± 0.023^{a}	0.001
22:0	0.762 ± 0.075	0.713 ± 0.041	0.781 ± 0.049	0.802 ± 0.038	0.767
22:1n-11	0.061 ± 0.011^{bc}	0.045 ± 0.005^{ab}	0.068 ± 0.005^{c}	0.031 ± 0.003^{a}	<0.0005
22:1n-9	0.151 ± 0.033^{ab}	0.125 ± 0.016^{a}	0.194 ± 0.018^{b}	0.098 ± 0.007^{a}	<0.0005
22:1n-7	0.042 ± 0.010^{a}	0.046 ± 0.007^{a}	0.053 ± 0.004^{b}	0.031 ± 0.003^{a}	0.001
22:4n-6	0.258 ± 0.079^{ab}	0.395 ± 0.076^{b}	0.323 ± 0.049^{b}	0.151 ± 0.023^{a}	<0.0005
22:5n-3	0.992 ± 0.239^{a}	1.156 ± 0.119^{a}	1.583 ± 0.114^{b}	0.901 ± 0.059^{a}	<0.0005
22:6n-3	0.611 ± 0.186^{b}	0.795 ± 0.117^{b}	1.055 ± 0.093^{c}	0.268 ± 0.017^{a}	<0.0005
24:0	0.325 ± 0.040^{bc}	0.357 ± 0.027^{b}	0.518 ± 0.054^{c}	0.172 ± 0.019^{a}	<0.0005
24:1n-9	0.141 ± 0.051^{bc}	0.079 ± 0.014^{b}	0.137 ± 0.016^{c}	0.037 ± 0.003^{a}	<0.0005
SFA	59.314 ± 1.028^{b}	59.510 ± 0.657^{b}	55.183 ± 0.499^{a}	60.547 ± 0.265^{b}	<0.0005
MUFA	32.767 ± 0.536	31.918 ± 0.278	31.578 ± 0.393	32.463 ± 0.159	0.219
PUFA	6.710 ± 0.823^{ab}	7.390 ± 0.546^{b}	11.041 ± 0.749^{c}	5.600 ± 0.204^{a}	<0.0005
n-6 PUFA	4.037 ± 0.417^{ab}	4.332 ± 0.279^{b}	6.981 ± 0.609^{c}	3.140 ± 0.113^{a}	<0.0005
n-3 PUFA	2.672 ± 0.442^{a}	3.058 ± 0.284^{a}	4.061 ± 0.219^{b}	2.460 ± 0.127^{a}	<0.0005
n-3/n-6 PUFA	0.647 ± 0.056^{ab}	0.670 ± 0.030^{a}	0.663 ± 0.038^{a}	0.799 ± 0.043^{b}	0.021

UFA/SFA	0.671 ± 0.031[a]	0.669 ± 0.018[a]	0.778 ± 0.016[b]	0.630 ± 0.006[a]	<0.0005
DMA	1.209 ± 0.097[a]	1.182 ± 0.082[a]	2.198 ± 0.102[b]	1.391 ± 0.079[a]	<0.0005
DBI	0.568 ± 0.037[ab]	0.594 ± 0.023[b]	0.739 ± 0.028[c]	0.513 ± 0.007[a]	<0.0005
TACL	17.068 ± 0.037[bc]	17.112 ± 0.018[c]	17.059 ± 0.032[b]	16.938 ± 0.011[a]	<0.0005

i = iso, *ai* = anteiso, DMA = dimethyl acetal, *i.e.*, plasmalogen alkenyl chain derivative, SFA = saturated fatty acid, MUFA = monounsaturated fatty acid, PUFA = polyunsaturated fatty acid, UFA = unsaturated fatty acid (MUFA + PUFA), DBI = double bond index, TACL = total average chain length

Dissimilar superscript letters indicate significant differences between sample types within a row (Kruskal–Wallis ANOVA, $p < 0.05$)

Table S2. Primer sequences of the studied genes.

Gene		Sequence (5'−3')	Length
RPLP0	FOR	5' AGATGCAGCAGATCCGCAT 3'	319 bp
	REV	5' GTGGTGATACCTAAAGCCTG 3'	
HAS1	FOR	5' CAAGATTCTTCAGTCTGGAC 3'	124 bp
	REV	5' TAAGAACGAGGAGAAAGCAG 3'	
HAS2	FOR	5' CAGAATCCAAACAGACAGTTC 3'	187 bp
	REV	5' TAAGGTGTTGTGTGTGACTG 3'	
HAS3	FOR	5' CTTAAGGGTTGCTTGCTTGC 3'	194 bp
	REV	5' GTTCGTGGGAGATGAAGGAA 3'	
CD44	FOR	5' CATCTACCCCAGCAACCCTA 3'	153 bp
	REV	5' CTGTCTGTCCTGTCGGTGAT 3'	
IL-1β	FOR	5' TGGAGCAACAAGTGGTGT 3'	157 bp
	REV	5' TTGGGATCTACACTCTCCAGC 3'	
IL-6	FOR	5' TGCAATAACCACCCCTGACC 3'	163 bp
	REV	5' GTGCCCATGCTACATTTGCC 3'	
TNF-α	FOR	5' CCCAGGGACCTCTCTCTAATCA 3'	80 bp
	REV	5' GCTACAGGCTTGTCACTCGG 3'	
COX-2	FOR	5' ATCTACGGTTTGCTGTGGGG 3'	156 bp
	REV	5' GCCTGCTTGTCTGGAACAAC 3'	
PPAR-α	FOR	5' GCTGTCACCACAGTAGCTTG 3'	197 bp
	REV	5' TTCCAGAACTATCCTCGCCG 3'	
PPAR-γ	FOR	5' TTCTCAAACGAGAGTCAGCCT 3'	126 bp
	REV	5' CAAAGGAGTGGGAGTGGTCT 3'	

RPLP0 = ribosomal protein lateral stalk subunit P0, *HAS1–3* = hyaluronan synthase 1–3, *CD44* = cluster of differentiation 44, *IL-1β/6* = interleukin-1*β*/6, *TNF-α* = tumor necrosis factor *α*, *COX-2* = cyclooxygenase 2, *PPAR-α/γ* = peroxisome proliferator-activated receptor *α/γ*
